# Diurnal Variations in Photochemical Energy Utilization and Osmotic Adjustments in Black Poplar Leaves Under Progressive Water Stress

**DOI:** 10.1111/pce.70135

**Published:** 2025-08-21

**Authors:** Antonella Gori, Mauro Centritto, Anatoly P. Sobolev, Giovanni Marino, Francesco Loreto, Francesca Alderotti, Cecilia Brunetti

**Affiliations:** ^1^ Department of Agri‐Food Production and Environmental Sciences (DAGRI) University of Florence Florence Sesto Fiorentino Italy; ^2^ National Research Council of Italy Institute for Sustainable Plant Protection (IPSP) Florence Sesto Fiorentino Italy; ^3^ National Research Council of Italy, Institute for Biological Systems “Annalaura Serge” Magnetic Resonance Laboratory Monterotondo Italy; ^4^ Department of Biology University of Naples Federico II Naples Italy

## Abstract

Drought limits the productivity of fast‐growing woody crops, although the metabolic adjustments conferring water stress tolerance remain poorly understood. We investigated the responses of *Populus nigra* seedlings to water stress by integrating daily physiological measurements and NMR metabolomic analyses. Our aims were to: (i) determine key metabolic and biochemical responses in leaves subjected to moderate (WS1) and severe (WS2) water stress and (ii) identify the metabolites responsible for dissipating excess photochemical energy and maintaining cellular turgor. Despite the reduction in electron transport rate, photoprotective mechanisms were activated. A threefold increase in isoprene emission was observed at midday in WS1 plants, while ascorbic acid and other antioxidants were higher under WS2 conditions. Relative water content and osmotic potential decreased throughout the day, suggesting that passive osmotic adjustments were primarily driven by soluble sugar accumulation. Organic acid reduction in WS1 (−9%) and WS2 (−17%) plants suggested an inhibition of the tricarboxylic acid cycle. Additionally, amino acids were twofold higher in water‐stressed plants compared to controls, likely reflecting an increased demand for primary and secondary metabolite biosynthesis. Our results provide new insights into the daily response of *P. nigra* to water stress, highlighting a delicate balance between metabolic and physiological adjustments.

## Summary statement

- Photoprotective mechanisms activated in WS plants, mainly involved isoprene emission and ascorbic acid.
- Metabolomic results suggest an inhibition of the tricarboxylic acid cycle and an higher metabolic demand for primary and secondary metabolites synthesis in WS than WW plants.

## 1 Introduction

Drought poses significant challenges for woody crops, impacting their growth and productivity (Centritto, Tognetti, et al. 2011). As climate change leads to more frequent and intense drought events, understanding the effects of water scarcity on woody crops becomes increasingly important for sustainable agriculture and forestry practices.

As sessile and long‐living organisms, trees respond dynamically to drought conditions, by making both long‐ and short‐term physiological and biochemical adjustments throughout the day (Gori et al. 2019; Robertson et al. 2022; Sow et al. 2021).

At the physiological level, drought causes detrimental effects on plant–water relations and photosynthesis. Drought‐induced reduction in leaf water potential primarily leads to the progressive closure of stomata, which is essential to minimize water loss through transpiration and prevent embolism formation (Brunetti et al. 2020); however, this mechanism unavoidably limits photosynthetic activity (Nadal‐Sala et al. 2024). Reduced photosynthesis often (especially at midday, under peaking temperatures and solar irradiance) leads to excess of reducing power built by the light‐harvesting complexes (Tattini et al. 2015; Perera‐Castro and Flexas 2023).

Biochemically, trees respond to drought by enhancing the biosynthesis of protective metabolites. These include (a) osmolytes (soluble carbohydrates, amino acids, amines, and polyols) which act both as compatible solutes, stabilizing cellular proteins and structures, and osmoprotectans, maintaining cell turgor under drought induced osmotic stress (Jia et al. 2020; Blake et al. 1984; Seki et al. 2007); (b) compounds and processes helping dissipate thermal energy and maintaining redox regulation. This may occur via activation of many enzymatic and nonenzymatic antioxidants, for example, (i) the xanthophyll cycle that is involved in the dissipation of excess excitation energy as heat (NPQ) (Qaderi et al. 2023; Demmig‐Adams and Adams 2006); (ii) the Hallywell–Asada‐Foyer enzymatic cycle to avoid the over‐reduction of the photosystems (Foyer and Noctor 2000); and (iii) secondary metabolites (e.g., polyphenols, isoprenoids) (Brunetti et al. 2015; Y. Sun and Fernie 2024).

Among these secondary metabolites, isoprene, a volatile organic compound (VOC), is also recognized for its pivotal role in shielding leaves of trees against the damage of abiotic stresses, particularly during episodes of high temperatures and drought stress (Centritto, Brilli, et al. 2011; Sharkey et al. 2008). Past research suggested that isoprene production influences the antioxidant system by neutralizing harmful radicals (Velikova et al. 2004, 2011; Vickers et al. 2009) or by strengthening and maintaining fluidity of photosynthetic membranes (Sharkey et al. 1996; Pollastri et al. 2019). Moreover, isoprene‐emitting plants rewire the entire proteome possibly signalling or priming defensive responses (Velikova et al. 2014) or even acting as a hormone‐regulator or hormone‐like substance (Dani and Loreto 2022). Furthermore, it has been shown that the capacity of emitting isoprene also affects biosynthesis of other defensive metabolites formed by the same pathway (the MEP pathway), such as carotenoids and abscisic acid (ABA) (Tattini et al. 2014). In particular, ABA is a key hormone involved in stomatal closure and in the regulation of drought‐responsive genes, whose leaf biosynthesis is strictly regulated in response to water stress (Cardoso et al. 2020).

Despite significant progress in understanding the physiological and biochemical responses of trees to water stress, a comprehensive understanding of the metabolic regulation, particularly on a daily timescale, remains lacking (Rolland et al. 2006; Shinozaki and Yamaguchi‐Shinozaki 2007; Tattini et al. 2015). Furthermore, previous studies investigating the relationship between tree metabolites and physiology under drought have predominantly focused on individual compounds or specific metabolite classes rather than studying large‐scale metabolic changes (Sardans and Peñuelas 2013). This limitation can now be addressed through nontarget metabolomics, a powerful technology that enables a holistic understanding of plant metabolism (Arbona et al. 2013; Allwood et al. 2021). In this context, untargeted metabolomics based on ¹H NMR spectroscopy offers a robust approach that enables the detection of a broad range of relatively abundant metabolites, providing both a snapshot of the plant's chemical profile at a given time and insights into the main metabolic pathways activated under drought conditions (Noleto‐Dias et al. 2023).

The European black poplar (*Populus nigra* L.) is an economically important species renowned for its rapid growth and diverse ornamental and industrial applications, including pulp production, timber and biofuel (G. Taylor 2002; Stettler et al. 1996). The high growth rate and productivity of this species, and poplars in general, are linked to significant water requirements (Rosso et al. 2023). As climate change leads to increasingly drier environments, the sustainability and feasibility of poplar cultivation may be challenged unless drought tolerant or resilient genotypes are identified or developed through biotechnological approaches. In this regard, understanding the daily dynamics of foliar metabolic responses to water stress may be important to identify functionally relevant metabolic traits serving as biomarkers for the selection of drought‐tolerant genotypes in targeted breeding programmes (Rosso et al. 2023).

In this study, we expanded on our previous research (Brunetti et al. 2019) by adopting a comprehensive approach that combines daily physiological and biochemical analyses to examine the foliar metabolome of black poplar seedlings subjected to progressively increasing water stress, ranging from moderate to severe stress conditions. We aimed to identify key metabolic pathways associated with stress tolerance as drought severity intensifies through daily high‐throughput measurements. Specifically, we integrated physiological phenotyping with metabolomics to:

1. Identify, using NMR, the leaf metabolites involved in dissipating excess of photochemical energy under water stress.
2. Detect metabolites that contribute to maintaining cellular turgor and homoeostasis under drought conditions.

## 2 Materials and Methods

### 2.1 Plant Material and Experimental Conditions

One‐year‐old cuttings of *P. nigra* L. were cultivated outdoors in Sesto Fiorentino, Italy (43°49′N, 11°37′), in 20 dm^3^ pots. In August 2017, plants were assigned to three water treatments: moderate water stress (WS1, 4 plants), severe water stress (WS2, 4 plants) and well‐watered (WW, 8 plants), the latter serving as controls. Water stress was induced by withholding irrigation for 4 days (WS1) and 8 days (WS2), while WW plants were irrigated daily to maintain pot capacity throughout the 8‐day experimental period (from 28 August to 4 September 2017). As previously described in Brunetti et al. (2019), moderate and severe water‐stress conditions were defined based on stomatal conductance (*g*_s_, mmol m^−2^ s^−1^), which decreased to approximately 32% (Day 4) and 6% (Day 8), of the values recorded in WW plants at 09:00 h. Physiological measurements (chlorophyll fluorescence and water relations) and sample collection for biochemical analyses (isoprene emission and NMR metabolomics) were conducted on Day 4 for WS1 plants, on Day 8 for WS2 plants and on both days for WW plants. Additional information on climatic conditions and the experimental setup are described in Brunetti et al. (2019).

### 2.2 Physiological Measurements

#### 2.2.1 Chlorophyll Fluorescence Measurements

Chlorophyll fluorescence measurements were performed on two fully developed leaves from four plants per treatment (*n* = 4) at three time points: 09:00, 13:00 and 18:00 h (solar time). At each time point, the two individual leaf measurements per plant were averaged to create a biological replicate. The actual photochemical efficiency of Photosystem II (Φ_PSII_) was assessed using a 2 cm^2^ cuvette at ambient CO_2_ concentration (~400 μmol mol^−1^) and 25°C with a portable infrared gas analyser (LI‐6400, Li‐Cor, Lincoln, NE, USA) equipped with a fluorimeter. The photosynthetic photon flux density (PPFD) was set to 1000 μmol m^−2^ s^−1^ at 09:00 h, 1800 µmol m^−2^ s^−1^ at 13:00 h and 1000 µmol m^−2^ s^−1^ at 18;00 h to simulate natural daily PPFD variation. The electron transport rate (ETR) was calculated following the method described in Genty et al. (1989) using the equation:

$$\text{ETR}\;=\Phi_{\text{PSII}}\times \text{PPFD}\times \alpha \times \beta ,$$

where *β* is the partitioning factor between photosystems I and II (~0.5) and *α* is leaf absorbance (~0.84). Furthermore, the ratio ETR/*P*_*n*_ (calculated using *P*_*n*_ values reported in Brunetti et al. 2019) was used as an indicator of excess electron transport that can be diverted toward alternative electron sinks rather than being utilized for photosynthesis and photorespiration.

Fluorescence parameters were also measured for the same leaves after a 30‐min dark‐adaptation. Following dark‐adaptation, minimal fluorescence (*F*_0_) was measured using weak modulated irradiation (< 1 μmol m^−2^ s^−1^). A 600‐ms saturating flash (> 7000 μmol m^−2^ s^−1^) was then applied to determine the maximum chlorophyll fluorescence yield (*F*_m_). Subsequently, *F*_m_′ was measured using another saturation flash (> 6000 μmol m^−2^ s^−1^), and non‐photochemical quenching (NPQ) was calculated as NPQ = (*F*_m_ – *F*_m_′)/(*F*_m_′) (Genty et al. 1989).

#### 2.2.2 Water Relations

To evaluate the impact of water stress on leaf water status, two leaves per plant from each treatment were collected daily at four specific time points: predawn (05:00), 09:00, 13:00, and 18:00 h. Leaves were placed in pre‐weighed (tared) zip‐locked plastic bags and stored in a refrigerator to preserve their condition during transportation to the laboratory. Leaves were promptly weighed after cutting, to determine their fresh mass (FW), and then allowed to hydrate overnight to determine turgid mass (TW) the following day. Finally, leaves were dried at 80°C for 48 h to determine their dry mass (DW). Relative water content (RWC, %) was calculated using the formula:

RWC(%)= (FW−DW)/(TW−DW).

Leaf osmotic potential (*Ψ*_π_) was measured using a Wescor VAPRO 5520 osmometer (Wescor Inc., Logan, UT) based on the boiling point method, using the same leaves previously measured for water potential (see Brunetti et al. 2019). Individual RWC and *Ψ*_*π*_ measurements were combined to generate a biological replicate per plant (*n* = 4).

The contribution of each metabolite, identified through NMR analysis, to leaf osmotic potential (*π*_*x*_) was determined using the following formula (Nobel 1999):

$$\pi_{x}(\%)=(\text{RT × RDW ×}\;C_{x})/\Psi_{\pi },$$

where *π*_*x*_ indicates the contribution of each individual compound to *Ψ*_*π*_, RDW is the relative dry weight at saturation (kg m^−3^), calculated considering the TW and DW of leaf RWC (see the previous section), *C*_*x*_ is the molar concentration of each metabolite (mol kg^−1^), and the RT value at 25°C is −0.002479 m^3^ MPa^−1^ mol^−1^. Finally, the percentage contribution of the different metabolite classes to *Ψ*_π_ was obtained as the sum of the contributions of individual compounds.

### 2.3 Biochemical Measurements

#### 2.3.1 Isoprene Analysis

The emission of isoprene was measured from the same leaves used for chlorophyll fluorescence analysis and on the same day. At the outlet of the Li‐Cor cuvette, a portion of the air (200 cm^3^ min^−1^) was diverted using an environmental air sampling device (A.P. BUCK Inc., Orlando, FL) into a silico‐steel cartridge containing 200 mg of Tenax adsorbent (Agilent, Cernusco sul Naviglio, Italy). A volume of 4 dm^3^ of air was pumped through the cartridges. Blank samples were taken using the same sampling procedure, but from empty cuvettes before enclosing the leaves, at each sampling time.

Next, the cartridges were desorbed using a thermal TurboMatrix unit (Perkin Elmer Inc., Waltham, MA, USA), and the analysis was performed using a Perkin Elmer Clarus 580 gas chromatograph equipped with a Clarus 560 Mass‐Selective‐Detector and a TurboMatrix thermal desorber (Perkin Elmer Inc., Waltham, MA, USA). The desorbed compounds were separated using a 30 m Elite‐5 MS capillary column, with the initial temperature set at 40°C for 5 min, followed by a temperature increase of 5°C min^−1^ up to 250°C, where it was held for 2 min. Helium was used as the carrier gas.

GC calibration was performed using an isoprene standard at various dilutions, starting from an initial concentration of 1000 ng g^−1^ (Rivoira, Milan, Italy). To verify the GC peak retention time, parent ions and main fragments in the spectra were analysed using the NIST library included in the GC/MS Turbomass software.

#### 2.3.2 NMR Leaf Metabolomic Analysis

For each treatment, three leaves from four plants were pooled and collected for metabolome analysis (*n* = 4) at four different harvesting times (5:00, 9:00, 13:00, 18:00 h). The collected leaves were immediately frozen in liquid nitrogen, stored at −80°C and subsequently lyophilized. Lyophilized and finely powdered leaf tissues (25 mg) were extracted using 1 mL of CH_3_CN/H_2_O mixture (1:1 v/v) and agitated for 1 min at room temperature. Following extraction, the samples were clarified by centrifugation (6700*g* for 4 min), and the resulting supernatant was dried under an N_2_ flux. The dry residue was then dissolved in 0.7 mL of 400 mM D_2_O phosphate buffer (pH = 6.5), containing 1.0 mM of 3‐(trimethylsilyl)propionic‐2,2,3,3‐d4 acid sodium salt as an internal standard. The samples were transferred into a standard 5 mm NMR tube.

NMR spectra were recorded at 27°C on a Bruker AVANCE 600 spectrometer operating at a proton frequency of 600.13 MHz.

^1^H NMR spectra were acquired at 300 K by co‐adding 256 transients with a recycle delay of 7 s, a 45° flip angle pulse of 8.0 μs and 32 K data points. A long single soft pulse applied during the last 2 s of relaxation delay was used to suppress the residual HDO signal. Before Fourier transformation, a small line broadening factor (0.4 Hz) was applied, followed by baseline corrections. TSPA methyl group signal at *δ* = 0.00 ppm was used as an internal reference.

2D NMR experiments, namely ^1^H‐^1^H TOCSY, ^1^H‐^13^C HSQC and ^1^H‐^13^C HMBC, were carried out under the experimental conditions previously reported. The mixing time for the ^1^H–^1^H TOCSY was 80 ms. The ^1^H‐^13^C HSQC experiments were performed with polarization transfer delays Δ of 1.66 ms (based on ^1^ *J*_C–H_ of 150 Hz). For ^1^H‐^13^C HMBC experiments, a delay of 80 ms was applied for the evolution of long‐range couplings.

Bruker TOPSPIN software version 1.3 was utilized for spectra processing. The integrals of selected signals (Table 1) were measured and normalized relative to the TSPA integral (at 0.00 ppm), which was set to 100. Metabolite concentrations (expressed in μmol/g dry weight, DW) were calculated using TSPA as an internal standard, based on the proportionality between integrals and molar concentrations.

**Table 1:** Relative water content (RWC) and osmotic potential (*Ψ*_*π*_), in well‐watered plants (WW), water‐stressed plants (WS1) and severely water‐stressed plants (WS2) at four time points: 5:00, 09:00, 13:00 and 18:00 h (solar time).

Treatment	Time	RWC (%)	*Ψ*_*π*_ (MPa)
WW	5:00	99.01 ± 1.31a	−1.56 ± 0.08a
	9:00	96.86 ± 1.73a	−1.84 ± 0.03b
	13:00	95.33 ± 2.55a	−2.16 ± 0.13c
	18:00	94.78 ± 4.51a	−2.28 ± 0.22c
WS1	5:00	94.46 ± 1.97a	−1.89 ± 0.05b
	9:00	90.60 ± 2.86b	−2.03 ± 0.03c
	13:00	86.42 ± 2.60c	−2.03 ± 0.03c
	18:00	93.55 ± 1.92b	−1.95 ± 0.03b
WS2	5:00	95.05 ± 2.07a	−1.80 ± 0.08b
	9:00	84.73 ± 1.12c	−1.75 ± 0.08b
	13:00	80.65 ± 1.02d	−2.03 ± 0.03c
	18:00	93.85 ± 1.27ab	−1.72 ± 0.09b
Significance			
Treat		**< 0.001**	**0.004**
Time		**< 0.001**	**< 0.001**
Treat × Time		**< 0.001**	**< 0.001**

*Note:* A two‐way ANOVA followed by Tukey's pairwise comparison was conducted to analyse the data, which are reported as means ± SD (*n* = 4). Significant *p*‐values (≤ 0.05) are in bold.

### 2.4 Statistics

Statistical analysis was performed using SPSS software programme (SPSS v.20; IBM, Chicago, IL, USA). For non‐destructive measurements (chlorophyll fluorescence and isoprene emission), a two‐way repeated‐measures ANOVA was applied, with water treatment as the between‐subjects factor and sampling time as the within‐subjects factor. For the destructive measurements (water relations and metabolomic analyses), a two‐way ANOVA was applied, with harvesting time and water treatment considered as two independent factors in a 4 × 3 between‐group design, representing four harvesting times and three water stress levels (WW, WS1, and WS2), respectively. Significant differences among means were determined at *p* < 0.05 using Tukey's post hoc test. Because no significant differences in physiological parameters were observed between WW samples collected on Day 4 and Day 8 (Supporting Information S1: Table SM1), biochemical analyses were not performed on WW samples from Day 8. Consequently, physiological and biochemical data are presented for WW and WS1 plants on Day 4 and for WS2 plants on Day 8.

## 3 Results

### 3.1 Physiological Responses

#### 3.1.1 Chlorophyll Fluorescence Parameters

**Figure 1:**
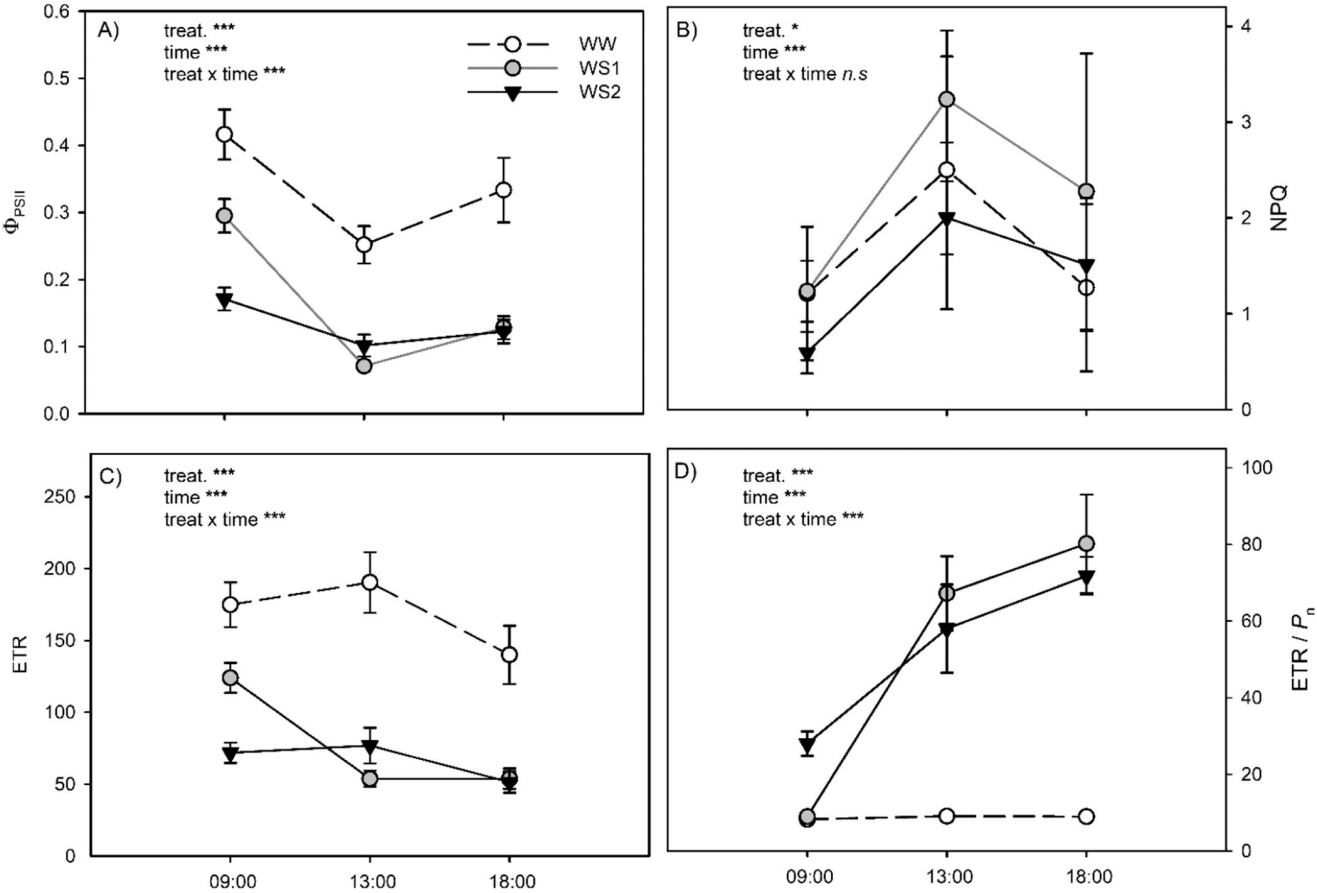
Chlorophyll fluorescence parameters in well‐watered (WW), moderate water‐stressed (WS1), and severely water‐stressed (WS2) plants at three time points: 09:00, 13:00, and 18:00 h (solar time). (A) Actual photochemical efficiency of Photosystem II (ΦPSII); (B) non‐photochemical quenching (NPQ); (C) electron transport rate (ETR); (D) electron transport rate and photosynthesis ratio (ETR/*P*_*n*_). A two‐way ANOVA followed by Tukey's post hoc test was used to analyse differences among treatments (means ± SD, *n* = 4). Detailed results of the two‐way ANOVA are provided in Supporting Information S1: Table SM2.

The actual efficiency of PSII (Φ_PSII_) showed a typical daily pattern, with a decline during the central hours of the day and higher values in the morning and late afternoon across all treatments (Figure 1A and Supporting Information S1: Table SM2). At 9:00, Φ_PSII_ values were significantly lower in WS2 plants compared to WW and WS1 plants (Figure 1A and Supporting Information S1: Table SM2). By 13:00, Φ_PSII_ markedly decreased in WS1 plants (−72%), and also declined in WW (~−30%) and in WS2 (~−40%) plants (Figure 1A). In the afternoon, Φ_PSII_ significantly increased from 13:00 to 18:00 in WW and WS1 plants, whereas no significant variation was observed in WS2 plants (Figure 1A and Supporting Information S1: Table SM2).

The midday depression in Φ_PSII_ values was associated with an increase in the NPQ of chlorophyll fluorescence across all treatments, which was largely relaxed in the late afternoon (Figure 1B).

The ETR was higher in WW plants compared WS1 and WS2, with distinct daily patterns among treatments. In WW and WS2 plants, ETR peaked at 13:00, although values were not statistically different from those recorded at 9:00. In WS1 plant, ETR values approximately halved from 9:00 to 13:00 and remained unchanged at 18:00 (Figure 1C and Supporting Information S1: Table SM1).

The ETR/*P*_*n*_ ratio remained unchanged in WW plants throughout the day, whereas it significantly increased in WS1 and WS2 plants at 13:00 and further at 18:00 (Figure 1D and Supporting Information S1: Table SM2).

#### 3.1.2 Water Relations

RWC varied significantly across treatments, with WW plants showing higher RWC values compared to WS1 and WS2 plants (Table 1). Additionally, the daily RWC trend differed among treatments. In WW plants, RWC remained stable throughout the day, whereas in WS1 and WS2 plants, it significantly declined from 5:00 to 9:00, followed by a further reduction at 13:00, and final recovery at 18:00 (Table 1).

Leaf osmotic potential (*Ψ*_*π*_) showed a significant daily trend, irrespective of treatment (Table 1). In WW plants, *Ψ*_*π*_ showed a substantial decline throughout the day, reaching its lowest values at 18:00. In contrast, in WS1 and WS2 plants had lower *Ψ*_*π*_ values than WW plants at the beginning of the day, with a further significant declined occurring only during the central hours (9:00–13:00, Table 1).

### 3.2 Metabolic Responses

#### 3.2.1 Isoprene Emission

**Figure 2:**
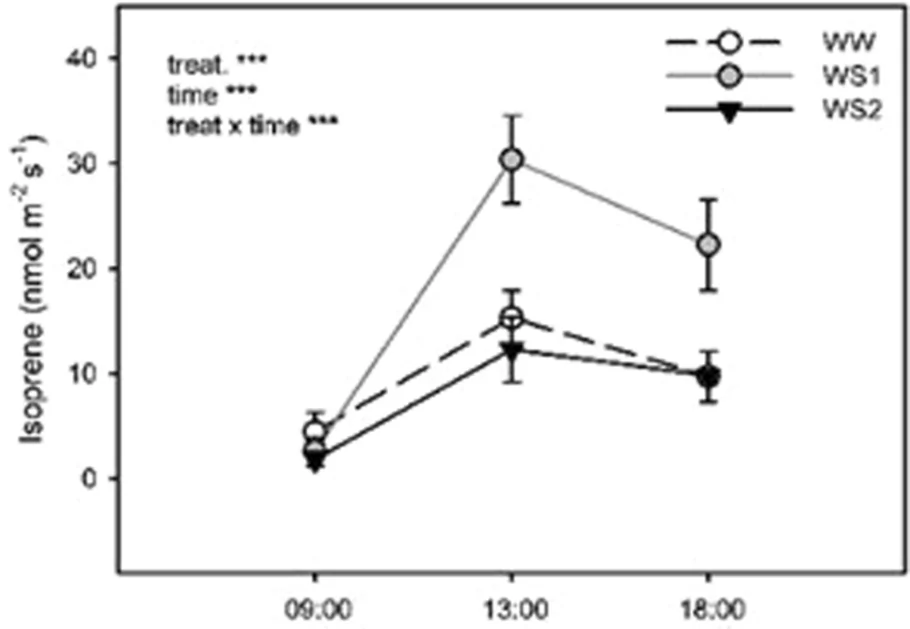
Isoprene emissions in well‐watered plants (WW), water‐stressed plants (WS1) and severely water‐stressed plants (WS2) at three time points: 09:00, 13:00 and 18:00 h (solar time). A two‐way ANOVA followed by Tukey's pairwise comparison was performed to analyse the data, presented as means ± SD (*n* = 4). Detailed results of the two‐way ANOVA are provided in Supporting Information S1: Table SM3.

Isoprene emission showed a distinct daily pattern across all treatments, increasing from morning to midday and declining in the afternoon. This increase was particularly pronounced in response to moderate water stress, showing a 3–4‐fold rise from 9:00 to 13:00 (Figure 2 and Supporting Information S1: Table SM3).

**Figure 3:**
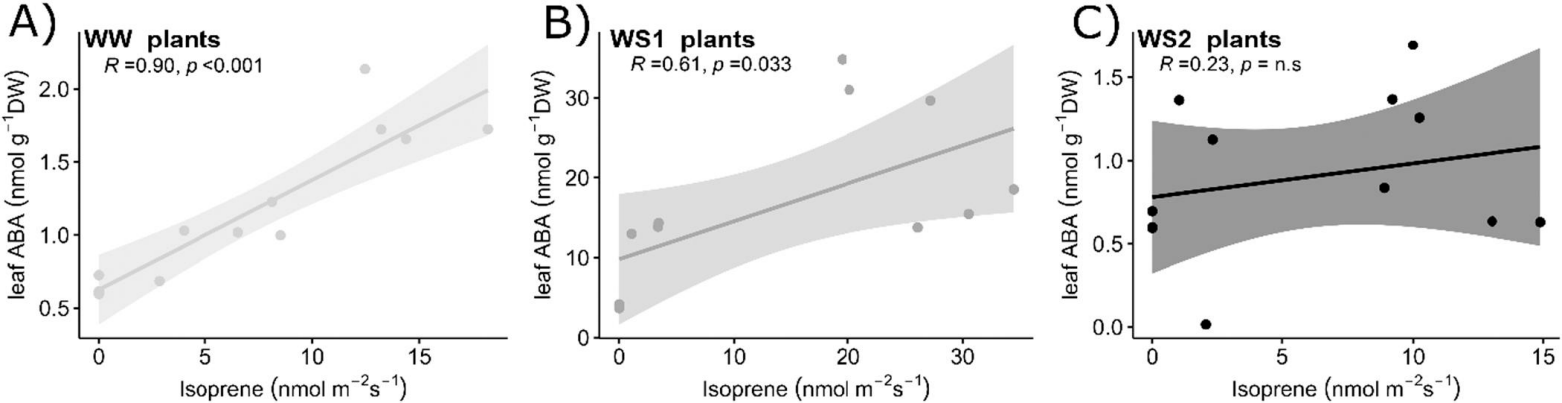
Pearson correlations between leaf abscisic acid (ABA) concentrations (data from Brunetti et al. 2019) and isoprene emission were evaluated in (A) well‐watered (WW), (B) moderately water‐stressed (WS1) and (C) severely water‐stressed (WS2) plants.

Daily data of isoprene emission (from 5:00 to 18:00) showed a strong and positive correlation with leaf ABA concentration (data from Brunetti et al. 2019) in WW and WS1 plants, which was not observed in WS2 plants (Figure 3). In addition, this correlation strengthened considering data of isoprene emission and leaf ABA concentration from 5:00 to 13:00 in WW and WS1 plants (Supporting Information S1: Figure SM3).

#### 3.2.2 NMR‐Based Leaf Metabolomics Profiling

A total of 22 metabolites, classified into four classes of compounds (amino acids, sugars, organic acids and phenolics), were identified in *P. nigra* leaf aqueous extracts. The average ^1^H NMR spectrum was dominated by sugar signals in the 5.5–3.0 ppm range. Additionally, intense signals in the 8.0–6.0 ppm spectral range indicated a relatively high content of aromatic compounds (amino acids and phenols) (Supporting Information S1: Figure SM1).

A total of eight amino acids were identified, including valine (Val), threonine (Thr), glutamine (Gln), asparagine (Asn), choline (Chol), arginine (Arg), glutamic acid (Glu) and alanine (Ala). Among the organic acids detected in the extracts (ascorbic, citric, malic, quinic and succinic), quinic acid was the most abundant. Furthermore, the NMR investigation revealed the presence of several phenolic compounds, namely pyrocatechol, salicin, salicyl alcohol, salicortin and rutin. Lastly, the NMR signals of three main sugars (glucose, fructose and sucrose) were assigned considering their various anomeric forms (α‐ and β‐anomers in the case of glucose, β‐fructopyranose and fructofuranose as the most abundant anomers in the case of fructose). Additionally, the spectra displayed relatively intense signals attributed to the sugar alcohol myo‐inositol.

**Figure 4:**
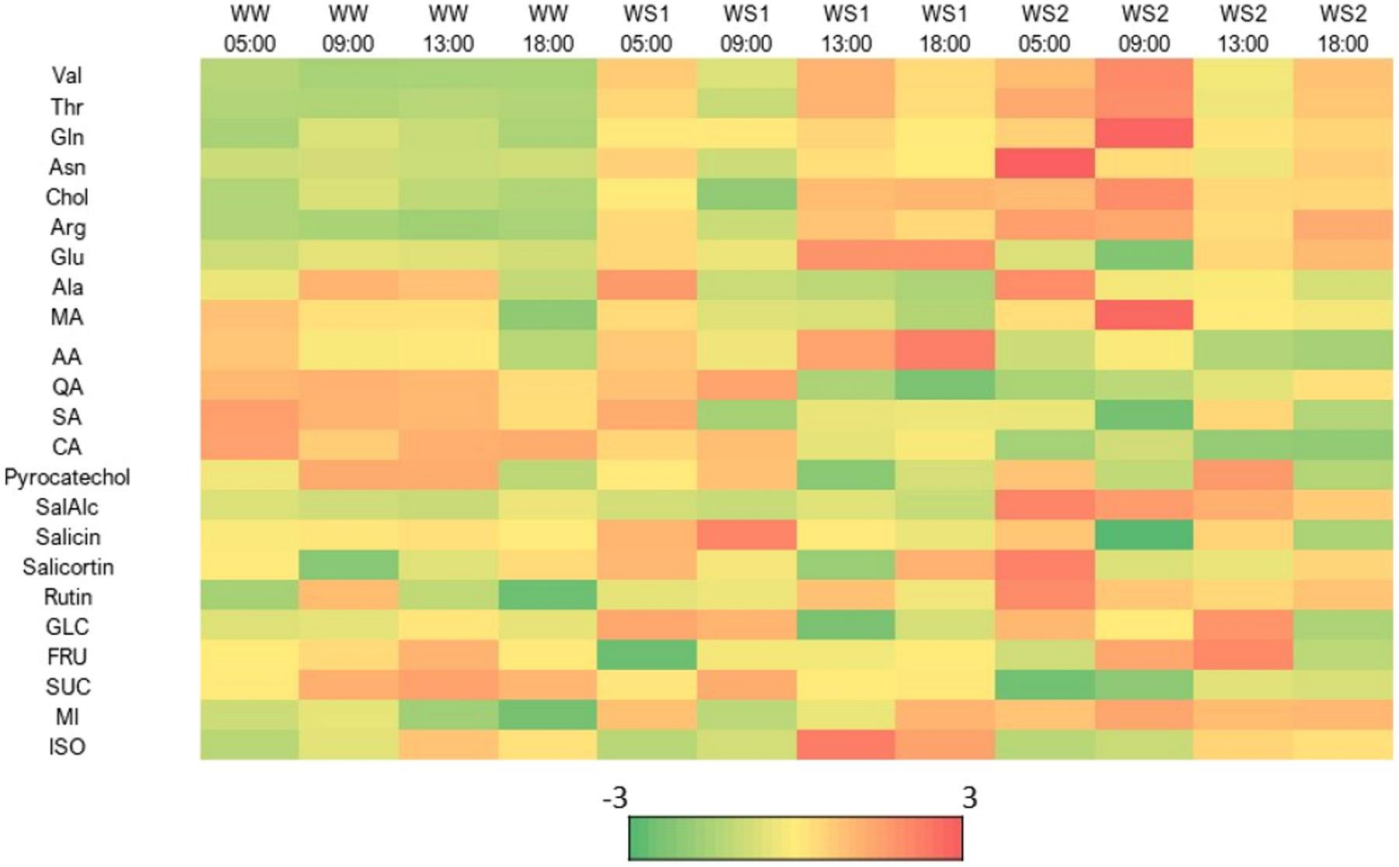
Heat map of metabolites identified through NMR metabolomics in well‐watered plants (WW), water‐stressed plants (WS1) and severely water‐stressed plants (WS2) at four time points: 5:00, 09:00, 13:00 and 18:00 h (solar time). Metabolite concentrations are colour‐coded on a normalized scale ranging from a minimum of −3 (green) to a maximum of 3 (red). AA, ascorbic acid; Ala, alanine; Arg, arginine; Asn, asparagine; CA, citric acid; Chol, choline; FRU, fructose; Gln, glutamine; GLC, salicin, salicortin, rutin, glucose; Glu, glutamic acid; ISO, isoprene; MA, malic acid; MI, myo‐inositol; QA, quinic acid; SA, succinic acid; SalAlc, pyrocatechol, salicyl alcohol; SUC, sucrose; Thr, threonine; Val, valine.

Results from the two‐way ANOVA analysis (Supporting Information S1: Table SM4) indicated that water stress treatments significantly affected the levels of 18 out of 22 metabolites (*p* < 0.05). Specifically, both WS1 and WS2 plants showed markedly higher amino acid content (except for Ala), while organic acid content showed only minor variations among treatments (Supporting Information S1: Tables SM5A and SM6A and Figure 4). Among the phenolic compounds, salicyl alcohol and rutin were significantly higher in WS2 plants compared to both WW and WS1 plants (Supporting Information S1: Table SM7A). Additionally, WS1 and WS2 plants showed higher levels of glucose and myo‐inositol but reduced sucrose levels relative to WW plants (Supporting Information S1: Table SM8A). In addition, water treatment induced a decline in sucrose content, with the lowest values observed in WS2 plants (Supporting Information S1: Table SM8A).

The time of the day significantly influenced the levels of 11 out of the 22 metabolites (*p* < 0.05) (Supporting Information S1: Table SM4). Specifically, five amino acids (Gln, Asn, Arg, Glu and Ala), two organic acids (malic and succinic acid), two phenolic compounds (salicortin and rutin) and three sugars (glucose, sucrose and fructose) showed significant daily fluctuations. These variations are also visually represented in the heatmap (Figure 4). In WS2 plants, glutamine levels approximately doubled from 5:00 to 9:00 before returning to predawn values (Figure 4 and Supporting Information S1: Table SM5B). Asparagine peaked at 5:00, then sharply declined until 13:00, reaching its minimum value, followed by a slight increase at 18:00 (Figure 4 and Supporting Information S1: Table SM5B). Arginine levels in WS2 plants significantly decreased from morning to midday, then increased at 18:00 (Figure 4 and Supporting Information S1: Table SM5B). Glutamic acid markedly increased in WS1 and WS2 plants between 9:00 and 13:00 and remained stable until 18:00 (Figure 4 and Supporting Information S1: Table SM5B). Lastly, Alanine in WS1 and WS2 plants reached its peak at 5:00, followed by a significant decline throughout the day (Figure 4 and Supporting Information S1: Table SM5B).

Regarding organic acids, malic acid showed a decreasing trend in WW and WS1 plants after 5:00, whereas in WS2 plants, its levels doubled between 5:00 and 9:00 before returning to predawn values at 13:00 (Figure 4 and Supporting Information S1: Table SM6B). Succinic acid peaked at 5:00 in WS1 plants, whereas in WS2 plants, it reached its highest values at 13:00 (Figure 4 and Supporting Information S1: Table SM6B). In WS2 plants, salicortin values declined during the central hours of the day (9:00 and 13:00) and were highest at PD and 18:00 (Figure 4 and Supporting Information S1: Table SM7B). Rutin showed peak values at 9:00 in WW plants, while in WS2 plants, the highest contents were recorded at 5:00, followed by a progressive decline throughout the day (Figure 4 and Supporting Information S1: Table SM7B).

Regarding sugars, glucose content in WS1 plants significantly declined between 9:00 and 13:00, and then remained stable until 18:00. In contrast, in WS2 plants, glucose content increased from morning to 13:00 before decreasing again at 18:00 (Figure 4 and Supporting Information S1: Table SM7B). Sucrose content in WW plants significantly increased from 5:00 to 13:00, followed by a decline at 18:00. In WS1 plants, sucrose content showed a significant peak at 9:00, while WS2 plants showed minimum sucrose content in the early morning (5:00 and 9:00) and maximum levels at 13:00 and 18:00 (Supporting Information S1: Table SM8B). Fructose showed a significant increase only in WS2 plants at 13:00 (Figure 4 and Supporting Information S1: Table SM8B).

To investigate metabolic changes induced by water treatments during different daily conditions, principal component analysis (PCA) was performed, considering each time point as an individual and NMR‐ and isoprene‐derived metabolites as variables (Supporting Information S1: Figure SM2, Tables SM9 and SM10).

Metabolite profiles effectively differentiated WS2 plants from WW and WS1 plants through two principal components (PC1 and PC2), which explained 43% and 18% of the total variance, respectively. In particular, WW and WS2 plants were clearly separated, with sample clustering primarily driven by PC1. Additionally, WS1 and WS2 plants were distinguished based on a combination of PC1 and PC2 (Supporting Information S1: Figure SM2).

In general, the loading plots for metabolite data indicate that PC1 is positively driven by higher levels of amino acids (e.g., Thr, Val, Chol, Arg, Gln), phenolic compounds (salicortin, rutin), and myo‐inositol, while negatively driven by sucrose and citric acid (Supporting Information S1: Table SM9). PC2 is positively influenced by isoprene, ascorbic acid (AA), and glutamate, whereas glucose and alanine contribute negatively (Supporting Information S1: Figure SM2).

The PCA results showed that overall chemical differences among treatments were more pronounced than time course variations, except for WS1 samples harvested at 9:00, which clustered closely with WW samples. Specifically, WW samples from all time points showed similar metabolite profiles, clustering together in the negative direction of PC1. Additionally, Leaf chemical composition was similar at 13:00 and 18:00 in WS1 and at 9:00 and 18:00 in WS2, with these samples clustering together in the positive direction of PC1. However, leaves collected at predawn under WS1 and at 13:00 under WS2 were distinct from other samples along, separating along PC2 (Supporting Information S1: Figure SM2).

#### 3.2.3 Contribution of Different Metabolite Classes to Osmotic Adjustments

Not all metabolite classes contributed equally to osmotic potential, with soluble sugars having the highest influence on osmotic adjustments across all treatments, followed by organic acids (Table 2). In contrast, the contribution of amino acids on osmotic potential was minimal (Table 2).

**Table 2:** Percentage contribution of metabolite classes to osmotic potential in well‐watered plants (WW), water‐stressed plants (WS1), and severely water‐stressed plants (WS2) at four time points: 05:00, 09:00, 13:00 and 18:00 h (solar time).

Treatment	Time	Amino acids (%)	Organic acids (%)	Sugars (%)	Total (%)
WW	5:00	1.77 ± 0.51c	11.75 ± 1.91a	25.62 ± 3.94a	39.14 ± 6.19a
	9:00	1.81 ± 0.29bc	8.84 ± 0.84ab	24.58 ± 0.90a	35.23 ± 0.43a
	13:00	1.60 ± 0.06c	8.65 ± 1.49b	24.48 ± 2.60a	35.74 ± 4.06a
	18:00	1.35 ± 0.03c	8.15 ± 0.86b	23.59 ± 2.24a	33.09 ± 3.07ab
WS1	5:00	3.37 ± 0.44a	8.86 ± 1.31ab	21.81 ± 3.47ab	34.03 ± 4.55ab
	9:00	2.03 ± 0.07b	8.79 ± 1.31b	25.54 ± 1.39a	36.36 ± 2.57a
	13:00	3.44 ± 0.75a	6.38 ± 1.17b	16.27 ± 2.63b	26.10 ± 4.40b
	18:00	2.84 ± 0.20ab	6.00 ± 1.48b	17.75 ± 2.46b	26.59 ± 3.77b
WS2	5:00	3.57 ± 1.10a	7.88 ± 1.65b	13.17 ± 3.12c	24.62 ± 5.71b
	9:00	3.70 ± 1.15a	9.07 ± 2.91ab	15.34 ± 2.80bc	28.10 ± 6.83b
	13:00	1.87 ± 0.08c	7.07 ± 0.48b	17.86 ± 2.26bc	26.81 ± 2.81b
	18:00	3.69 ± 0.70a	8.42 ± 0.37b	17.15 ± 0.98bc	29.86 ± 1.89b
Significance					
Treatment		**< 0.001**	**0.016**	**< 0.001**	**< 0.001**
Time		0.218	**0.012**	0.301	0.181
Treatment × Time		**0.003**	0.163	**0.003**	0.68

*Note:* A two‐way ANOVA followed by Tukey's pairwise comparison was conducted to analyse the data, which are reported as means ± SD (*n* = 4). Significant *p*‐values (≤ 0.05) are in bold.

The role of soluble sugars in osmotic adjustment showed no significant diurnal pattern under WW and WS2 conditions. However, in WS1 plants, sugar contributions were notably higher in the morning (Table 2).

## 4 Discussion

The intensifying intensity and frequency of droughts caused by climate change can significantly affect plant productivity, particularly in fast‐growing species with high water demands, such as *P. nigra*. This study provides an in‐depth analysis of the physiological responses of black poplar to progressively increasing water stress, focusing on daily changes in plant metabolism, energy dissipation and osmotic adjustments.

Changes in chlorophyll fluorescence initially reflect adjustments in energy budget management, followed by impairment of PSII reaction centres and light‐harvesting complexes under water stress (Baker 2008). The observed midday increase in NPQ may be interpreted as the activation of reversible photoprotective mechanisms, which serve to dissipate excess excitation energy safely, thereby preventing photodamage. Specifically, NPQ is regulated by the xanthophyll cycle, which is closely linked to carbon flux through the MEP pathway (Brunetti et al. 2015). Thus, the increment of NPQ at 13:00, coinciding with the PPFD peak, across all treatments suggests a diurnal shift in xanthophyll cycle activity in response to environmental conditions (Supporting Information S1: Table SM2).

Diurnal ETR patterns varied across treatments. In WS1 plants, the midday decline likely reflects stress‐induced downregulation of photosynthesis. In contrast, ETR remained relatively stable in WS2, likely due to the very low photosynthetic activity under these conditions (data reported in Brunetti et al. 2019). Combining these results with photosynthetic rates, we may calculate an ETR/*P*_*n*_ ratio increasing to 60–80 in water‐stressed plants, consistently with Marino et al. 2017. This suggests the onset of photorespiration and the activation of alternative electron sinks under water stress (Perera‐Castro and Flexas 2023). Although the distribution of excess excitation energy is mainly diverted to photorespiration, a small proportion may stimulate the production of isoprene (Harrison et al. 2013; Grote et al. 2014), which likely mitigates oxidative stress, as suggested by previous studies (Fini et al. 2017). Isoprene emission is a characteristic feature of fast‐growing hygrophilous species such as *Populus* spp., serving as a crucial adaptive mechanism that enhances tolerance to acute, transient stress events (Loreto and Fineschi 2015). The peak emission of isoprene observed at midday coincides with periods of heightened environmental stress and this is in line with the role of this metabolite in mitigating oxidative damage to the photosynthetic apparatus (Velikova et al. 2011).

At the primary metabolism level, photorespiration effectively help dissipate excess reducing power through the electron transport chain (Wingler et al. 2000). Photorespiration is typically enhanced under stress conditions, such as water stress, where stomatal limitations reduce CO_2_ availability for photosynthetic fixation. This results in increased RuBisCO oxygenation over carboxylation unless Rubisco is deactivated under severe water stress conditions (Noctor et al. 2002). Alternatively, excess excitation energy can be dissipated via the Mehler reaction, where electrons from Photosystem II are directly transferred to O_2_ molecules. While these metabolic pathways effectively protect the photosynthetic apparatus against photoinactivation, they also generate the formation of reactive oxygen species (ROS), which necessitates scavenging by enzymatic antioxidants and secondary metabolites with antioxidant functions (Barchet et al. 2014). During the central hours of the day, secondary metabolites have been reported to play a more significant role as antioxidants than antioxidant enzymes in protecting woody plants against thermal and water stress (Tattini et al. 2015).

Our metabolite analysis suggests that moderately water‐stressed poplar plants activated both secondary metabolism (e.g., isoprene formation) and low molecular weight antioxidants, such as AA, for antioxidant defence. AA is known to protect membranes by scavenging oxygen‐free radicals and regenerating tocopherol from tocopheroxyl radicals under both water stress and high radiation conditions (Mishra et al. 2023). The higher AA content observed at 13:00 and 18:00 in WS1 may be attributed to the activation of l‐galactose dehydrogenase (l‐GalLDH) via the alternative cytochrome *c* reduction pathway (ACR pathway), highlighting the link between AA synthesis and mitochondrial respiration (Matos et al. 2022). Conversely, the elevated rutin concentrations found in WS2 compared to WS1 and WW plants suggest that severely water‐stressed plants experience increased oxidative stress, requiring secondary metabolites, such as phenylpropanoids and in particular dihydroxy B‐ring‐substituted flavonoids, to complement the function of antioxidant enzymes as ROS scavengers (Agati et al. 2013; Ahmed et al. 2021).

Isoprene emission is a functionally important metabolic process that help protect photosynthetic membranes from temperature and radiation‐induced damage (Pollastri et al. 2019). It is well known that isoprene emission is resistant to mild water stress (Brilli et al. 2007; Centritto, Brilli, et al. 2011; Marino et al. 2017). The literature also shows that during the early stages of drought, when water stress is still moderate, isoprene emissions can even increase (Marino et al. 2017; Z. Sun et al. 2020). Similarly, in our experiment, isoprene biosynthesis increased under WS1. Although carbon costly, increased isoprene biosynthesis under moderate water‐stress helps maintain the MEP‐pathway active, providing protection through both membrane stabilization (Velikova et al. 2011) and ROS scavenging (Tattini et al. 2014).

It is also well established that as water stress progresses, especially when it becomes severe, isoprene emission declines significantly (Brilli et al. 2007; Centritto, Brilli, et al. 2011). This reduction is often attributed to a limited supply of carbon substrates, resulting from decreased photosynthesis under water stress (Sharkey and Yeh 2001; Brilli et al. 2007; Brunetti et al. 2019). Interestingly, under severe water stress, carbon flux may be redirected to support the biosynthesis of other essential, non‐volatile isoprenoids, such as carotenoids and the carotenoid‐derived hormone ABA, which play critical roles in stress response and photoprotection (Rasulov et al. 2014; Marino et al. 2017). Accordingly, we propose that the lower isoprene emission observed in WS2 plants, relative to WS1, may result from a metabolic shift favouring the preferential synthesis of non‐volatile isoprenoids under severe stress conditions (Beckett et al. 2012; Harrison et al. 2013; Z. Sun et al. 2020).

Figure 3A shows a highly significant linear relationship between free ABA concentrations (as reported in fig. 4A of Brunetti et al. 2019) and isoprene emission rates (Figure 2) measured at three time points during daily sampling in well‐watered control plants. This correlation reflects the parallel diurnal patterns observed for both compounds and provides further evidence that ABA is synthesized in leaves via the MEP pathway (Barta and Loreto 2006; Marino et al. 2017; Xu et al. 2020), which is fuelled by photosynthetically derived carbon. Under moderate water stress conditions, the linear relationship becomes less statistically significant, although the correlation coefficient remains high (Figure 3B). In WS1 plants, as shown in fig. 4A of Brunetti et al. (2019), ABA concentrations accumulate at the end of the day as a consequence of water stress, whereas isoprene emission declines in response to reduced photosynthesis caused by lower PPFD. Although the correlation is lower compared to control plants, it still indicates that photosynthetic carbon continues to support the biosynthesis of both isoprene and ABA in the leaves. In contrast, the relationship between free ABA concentrations and isoprene emission rates is completely lost in plants subjected to severe water stress (Figure 3C). As observed in WS1, ABA levels remain elevated throughout the day in WS2 plants, while isoprene emission declines, particularly toward the end of the day. Moreover, throughout the day, ABA is consistently and strongly induced by the stress, while isoprene emission is markedly suppressed in WS2 plants. These contrasting responses explain the absence of correlation between the two compounds, despite both being synthesized via the MEP pathway. This suggests that, under severe water stress, photosynthetically derived carbon may be preferentially allocated to ABA biosynthesis rather than to isoprene production.

Our results suggest that soluble sugars, particularly sucrose, play a role in osmotic adjustments in *P. nigra* leaves under moderate water stress, exceeding other primary metabolites such as amino acids and organic acids. However, as water stress progressed, the content of soluble sugars significantly decreased, reducing their contribution to osmoprotection from approximately 24% in WW plants to 20% in WS1 and 16% in WS2 plants. This finding contrasts with previous studies reporting an increase in leaf soluble sugars concentrations in water‐stressed poplar plants (Yin et al. 2005; Xiao et al. 2009). However, we cannot exclude that mineral elements such as K and Ca may have acted as osmotically active molecules at midday, contributing to osmotic adjustment as previously reported in Traversari et al. (2020). Similar to our findings, Regier et al. (2009) observed a decline in soluble sugar and starch concentrations in water‐stressed *P. nigra* compared to well‐watered plants, attributing this to drought‐induced inhibition of carbon assimilation. While this explanation remains plausible, we do not exclude the possibility that osmotic adjustment may have occurred in other plant organs, such as roots. Because roots are less capable of controlling water loss than leaves, they may develop additional defences against dehydration when exposed to low‐soil water availability. Additionally, the capacity for osmotic adjustment varies widely within the genus *Populus* and depends on the intensity and duration of water stress (Kim et al. 2022). For instance, Kim et al. (2022) investigated drought tolerance in *Populus alba *× *Populus davidiana* and *Populus davidiana* in a pot experiment and found no significant variation in soluble sugar concentrations until the sixth consecutive day of water restriction.

On a daily timescale, leaf osmotic potential (*Ψ*_*π*_) showed significant variations throughout the day across all treatments. However, the contribution of soluble sugars to osmotic potential varied significantly only in WS1 plants, peaking at 9:00, alongside higher levels of sucrose and glucose concentrations and increased photosynthesis, as described by Brunetti et al. (2019). This pattern suggests that soluble sugar concentrations closely followed the photosynthetic trend, suggesting a primarily passive osmotic adjustment driven by a reduction in RWC throughout the day. The resulting decrease in cell volume in leaf tissues under dehydration supports this mechanism (Turner 2018). This result is in line with previous investigations conducted on *Populus* spp. indicating that, in leaves subjected to water stress, osmotic adjustments are predominantly passive and resulting from dehydration of leaf tissue leading to a higher concentration of osmolytes, particularly soluble sugars (Traversari et al. 2018). Thus, our study supports the hypothesis that poplar leaves are not the primary site for active accumulation of osmolites when compared to other plant tissues, such as bark, xylem and roots (Traversari et al. 2020; Brunetti et al. 2019).

Additionally, the significant daily variations in the contribution of sucrose to osmotic adjustment in WS1 plants, which mirrored the diurnal variations in sugar content, with the lowest levels recorded at 13:00 and 18:00, may be linked to sugar export from the leaf via the phloem system (Zhang et al. 2014). In contrast, the high leaf sucrose concentrations observed in WS2 plants at 13:00 and 18:00 may indicate an impairment or downregulation of primary metabolic processes in severely water‐stressed leaves. This could result in the conversion of the limited assimilated carbon into sucrose, leading to its temporary accumulation in the leaf vacuole (Lemoine et al. 2013; Salvi et al. 2022). Alternatively, the relatively high sucrose levels in leaves may stem from a delay in sugar transport from the mesophyll to the phloem and subsequently to sink organs, potentially limiting plant growth (Bachet et al. 2014; Aluko et al. 2021).

During drought conditions, amino acids can function as osmotically active metabolites. While we observed an increase in leaf amino acids levels in WS1 and WS2 plants compared to WW plants, their overall contribution to osmotic potential was minimal. This is likely due to the absence of proline accumulation in water‐stressed *P. nigra* leaves, as proline is a key osmoprotectant amino acid in plants (Jouve et al. 2004; Xiao et al. 2008). Our findings are consistent with previous research reporting minimal proline accumulation in the leaves of other *Populus* species under drought, highlighting variability in drought tolerance traits across the *Populus* genus (Tschaplinski and Tuskan 1994; Gebre et al. 1997). Therefore, the increased amino acid contents observed under moderate and severe stress may be associated with other processes, such as nitrogen balance and protein breakdown (Barchet et al. 2014). Furthermore, the higher amino acid concentrations in WS1 and WS2 plants compared to WW plants may indicate a reduction in protein synthesis in response to water stress, potentially leading to decreased plant growth and biomass production (Lawlor and Cornic 2002).

Amino acids have also been proposed to contribute to additional processes that enhance drought tolerance (Charlton et al. 2008; Jia et al. 2020; Zhang et al. 2018). For instance, an increase in asparagine‐derived amino acids has been reported to enhance poplar plant tolerance to abiotic stresses, particularly under drought and nutrient deficient conditions (Kirma et al. 2012; Zhang et al. 2018).

Previous studies suggest that branched‐chain amino acids such as valine, leucine, and isoleucine play a fundamental role in dehydration tolerance (Pires et al. 2016), while specific amino acids, including serine, arginine, glutamine, and alanine, are crucial nitrogen sources for plant nutrition under water stress, potentially acting as reservoirs of metabolic precursors for rapid recovery after rewatering (Batista‐Silva et al. 2019). In addition, under drought conditions, amino acids may be utilized as alternative substrates for mitochondrial respiration, contributing to ATP production and compensating for reduced photosynthesis (Bowne et al. 2012; N. L. Taylor et al. 2004). However, the precise roles of amino acid metabolism and catabolism under drought conditions, including their daily fluctuations, remain poorly understood. Furthermore, significant daily variations in leaf amino acid content have been rarely documented, highlighting the need for further research in this field.

Similarly to amino acids, organic acids contributed only modestly to osmotic potential adjustments compared to sugars and sugar alcohols (Charlton et al. 2008; Hamanishi et al. 2015). We observed a general reduction in organic acids content under drought conditions, consistent with previous studies investigating the effects of water stress on the TCA cycle (Hamanishi et al. 2015; Jia et al. 2020). The metabolic activity of the TCA cycle is known to be influenced by drought, although the precise physiological roles of its constituent enzymes and metabolites remain unclear (Araújo et al. 2012). The decline in TCA cycle intermediates, such as citrate and succinate, under water stress suggests that TCA cycle activity may be inhibited by water deficit (You et al. 2019). This inhibition has been associated with reduced carbohydrate synthesis and suppressed mitochondrial respiration (Barchet et al. 2014).

It is worth noting that TCA cycle intermediates, such as succinic acid, play roles beyond the citric acid cycle, including their involvement in γ‐aminobutyric acid (GABA) metabolism, a non‐proteinogenic amino acid known to mediate responses to biotic and abiotic stress (Seifikalhor et al. 2019; Rabara et al. 2017). Despite the overall decrease in TCA intermediates in WS plants, malic acid showed an opposite trend. Malic acid has been reported to increase in concentration following mild stress periods (MacLennan et al. 1963; Hamanishi et al. 2015). This organic acid plays a crucial role in the final steps of the TCA cycle, where malate dehydrogenase (MDH) catalyses its reversible oxidation to oxaloacetate (OAA), facilitating cycle restart (Nunes‐Nesi et al. 2005; Hamanishi et al. 2015). Under drought stress, a reduced utilization rate of malic acid in sink tissues may lead to its accumulation, potentially due to suppression of NAD‐dependent MDH (Rzepka et al. 2009). However, the functions of malic acid extend beyond the TCA cycle, with previous studies suggesting its involvement in stomatal regulation in response to elevated CO_2_ and low water availability (Qi et al. 2021; Wilkinson and Davies 2002).

## 5 Conclusion

Our results suggest that progressing water stress led to the accumulation of excess excitation energy in photosystem II, prompting plants to activate various dissipation and scavenging mechanisms. At the primary metabolism level, photorespiration may have contributed to use excess reducing power, while secondary metabolites with antioxidant functions, such as AA in WS1 plants and the flavonol rutin in WS2 plants, were also elicited, particularly during the central hours of the day, when photoprotection demand was highest. Additionally, the observed increase in isoprene emission may have helped mitigate temperature‐ and light‐induced damage to the photosystems in moderately water‐stressed plants. However, this effect appeared reduced in severely water‐stressed plants, possibly due to limited photosynthetic substrate availability for isoprene synthesis.

The decrease in RWC of water‐stressed black poplar leaves occurred along with a diurnal reduction in osmotic potential, suggesting passive osmotic adjustments. Our analysis indicates that soluble sugars were the primary metabolites driving osmotic adjustment, while the increase in amino acid concentrations in water‐stressed plants may reflect a decline in protein synthesis or increased proteolysis to support mitochondrial respiration, secondary metabolite production and precursors supply for protein biosynthesis. The significant daily variation in leaf amino acid content has been rarely documented and warrants further investigation. Finally, water stress induced a general decline in leaf organic acid levels, suggesting inhibition of the TCA cycle. However, malic acid concentrations were significantly higher in water‐stressed plants than in well‐watered ones, indicating its possible involvement in stomatal closure, particularly during the central hours of the day. Overall, this study provides a comprehensive perspective on the daily biochemical adjustments in black poplar leaves to cope with progressively severe water stress.

In addition, it highlights the intricate balance between metabolic adjustments and physiological responses, suggesting that water stress induces subtle yet significant alterations in the circadian regulation of this water‐spending species. Understanding these mechanisms is essential for breeding and selecting genotypes with improved drought tolerance, as it allows identification of traits linked to better tolerance and survival under increasingly frequent and severe water stress conditions.

## Conflicts of Interest

The authors declare no conflicts of interest.

## Supporting information


**Figure SM1:** ¹H NMR spectra of leaf aqueous extracts from *Populus nigra* plants in (a) well‐watered (WW, black trace), (b) water‐stressed (WS1, blue trace), and (c) severely water‐stressed (WS2, red trace). **Figure SM2:** The PCA score plot of the NMR‐based leaf metabolomic analysis in well‐watered plants (WW), water‐stressed plants (WS1), and severely water‐stressed plants (WS2) at four time points (05:00 h, 09:00 h, 13:00 h, and 18:00 h, solar time) illustrates the metabolic variation among treatments. **Figure SM3:** Pearson correlations between leaf ABA concentration and isoprene emission across pooled data from well‐watered plants (WW), water stress plants (WS1) and severe water stress plants (WS2). **Table SM1:** Summary of the statistical analysis results for chlorophyll fluorescence parameters (actual photochemical efficiency of Photosystem II, ΦPSII;non‐photochemical quenching, NPQ: electron transport rate, ETR; and the ETR/photosynthesis ratio. **Table SM2:** Summary of the statistical analysis results for chlorophyll fluorescence parameters comparing well‐watered plants (WW), water‐stressed plants (WS1), and severely water‐stressed plants (WS2) at four time points: 05:00 h, 09:00 h, 13:00 h, and 18:00 h (solar time). **Table SM3:** Summary of the statistical analysis results for isoprene emissions comparing well‐ watered plants (WW), water stress plants (WS1) and severe water stress plants (WS2) at three time points: 09:00 h, 13:00 h, and 18:00 h (solar time). **Table SM4:** Summary of the statistical analysis results for NMR‐based leaf metabolomic data in well‐ watered plants (WW), water stress plants (WS1) and severe water stress plants (WS2) at four time points: 05:00 h, 09:00 h, 13:00 h, and 18:00 h (solar time). **Table SM5:** Quantification of amino acids identified by leaf NMR metabolomic profiling in well‐ watered plants (WW), water stress plants (WS1) and severe water stress plants (WS2) at four time points: 05:00 h, 09:00 h, 13:00 h, and 18:00 h (solar time). **Table SM6:** Summary of the statistical analysis results of organic acids identified by leaf NMR metabolomic profiling in well‐watered plants (WW), water stress plants (WS1) and severe water stress plants (WS2) at four time points: 5:00 h, 09:00 h, 13:00 h, and 18:00 h (solar time). **Table SM7:** Summary of the statistical analysis results of phenolic compounds identified by leaf NMR metabolomic profiling in well‐watered plants (WW), water stress plants (WS1) and severe water stress plants (WS2) at four time points: 5:00 h, 09:00 h, 13:00 h, and 18:00 h (solar time). **Table SM8:** Summary of the statistical analysis results for sugars identified by leaf NMR metabolomic profiling in well‐watered plants (WW), water stress plants (WS1) and severe water stress plants (WS2) at four time points: 5:00 h, 09:00 h, 13:00 h, and 18:00 h (solar time). **Table SM9:** Compositional Scree plot of the metabolites detected by NMR leaf metabolomic analysis. Each row reports the variance of the corresponding principal component. **Table SM10:** Specific loadings of the metabolites detected by NMR leaf metabolomic analysis.

## Data Availability

The data that support the findings of this study are available from the corresponding author upon reasonable request.
