# Supplementary material for: Diurnal Variations in Photochemical Energy Utilization and Osmotic Adjustments in Black Poplar Leaves Under Progressive Water Stress
Source: Plant Cell Environ. 2025 Aug 21;49(10):7590–603. doi: 10.1111/pce.70135 (PMC13551634; doi:10.1111/pce.70135)
Supplement: Supplementary file 1 [file PCE-49-7590-s001.docx]

SUPPLEMENTARY MATERIALS

**Fig. SM1.** ¹H NMR spectra of leaf aqueous extracts from *Populus nigra* plants in (a) well-watered (WW, black trace), (b) water-stressed (WS1, blue trace), and (c) severely water-stressed (WS2, red trace). The data presented correspond to a single leaf aqueous extract but are representative of experiments replicated four times on different leaves.

**Fig SM2.** The PCA score plot of the NMR-based leaf metabolomic analysis in well-watered plants (WW), water-stressed plants (WS1), and severely water-stressed plants (WS2) at four time points (05:00 h, 09:00 h, 13:00 h, and 18:00 h, solar time) illustrates the metabolic variation among treatments. The first two principal components of the compositional scree plot (Table SM8) accounted for 60.89% of the total variance, with the first component explaining 42.68% and the second component contributing an additional 18.21%. Abbreviations: Valine (Val), Threonine (Thr), Glutamine (Gln), Asparagine (Asn), Choline (Chol), Arginine (Arg), Glutamic acid (Glu), Alanine (Ala), Malic acid (MA), Ascorbic acid (AA), Quinic acid (QA), Succinic acid (SA), Citric acid (CA), Pyrocatechol, Salicyl alcohol (SalAlc), Salicin, Salicortin, Rutin, Glucose (GLC), Fructose (FRU), Sucrose (SUC), and Myo-inositol (MI).

**Fig SM3**. Pearson correlations between leaf ABA concentration and isoprene emission across pooled data from well-watered plants (WW), water stress plants (WS1) and severe water stress plants (WS2).

| *Φ*PS2 | WW on day 4 | WW on day 8 | Significance |
| --- | --- | --- | --- |
| 09:00 | 0.416±0.037 | 0.417±0.037 | n.s |
| 13:00 | 0.252±0.028 | 0.252±0.027 | n.s |
| 18:00 | 0.333±0.048 | 0.334±0.044 | n.s |
| **ETR** |  |  |  |
| 09:00 | 174.838±15.633 | 175.151±15.439 | n.s |
| 13:00 | 190.354±21.086 | 190.753±20.446 | n.s |
| 18:00 | 140.028±20.229 | 140.282±18.625 | n.s |
| **NPQ** |  |  |  |
| 09:00 | 1.121±0.697 | 1.306±0.772 | n.s |
| 13:00 | 2.501±0.723 | 2.513±1.236 | n.s |
| 18:00 | 1.273±0.872 | 1.301±0.667 | n.s |
| **ETR/*P*_n_** |  |  |  |
| 09:00 | 8.231±0.266 | 8.247±0.279 | n.s |
| 13:00 | 9.061±0.932 | 9.082±0.913 | n.s |
| 18:00 | 8.924±0.927 | 8.943±0.831 | n.s |
| **RWC (%)** |  |  |  |
| 05:00 | 99.01±1.31 | 98.34±0.73 | n.s |
| 09:00 | 96.86±1.73 | 95.95±1.33 | n.s |
| 13:00 | 95.33±2.55 | 95.14±2.48 | n.s |
| 18:00 | 94.78±4.51 | 94.36±2.89 | n.s |
| **Ψ**π (MPa) |  |  |  |
| 05:00 | -1.56±0.08 | -1.57±0.06 | n.s |
| 09:00 | -1.84±0.03 | -1.83±0.03 | n.s |
| 13:00 | -2.16±0.13 | -2.16±0.12 | n.s |
| 18:00 | -2.28±0.22 | -2.26±0.16 | n.s |

**Tab SM1.** Summary of the statistical analysis results for chlorophyll fluorescence parameters (actual photochemical efficiency of Photosystem II, ΦPSII;non-photochemical quenching, NPQ: electron transport rate, ETR; and the ETR/photosynthesis ratio. ETR/Pn), as well as water relations parameters (relative water content, RWC; and osmotic potential, Ψπ). Comparisons were made between well-watered plants (WW) on day 4 and day 8. Data are reported as means ± SD (n = 4) and were analyzed using Student’s t-tests.

| Treatment | Time | ***Φ*PS2** | **ETR** | **NPQ** | **ETR/*P*_n_** |
| --- | --- | --- | --- | --- | --- |
| WW | 09:00 | 0.416±0.037 a | 174.838±15.633 ab | 1.121±0.697 b | 8.231±0.266 d |
|  | 13:00 | 0.252±0.028 c | 190.354±21.086 a | 2.501±0.723 ab | 9.061±0.932 d |
|  | 18:00 | 0.333±0.048 b | 140.028±20.229 b | 1.273±0.872 ab | 8.924±0.927 d |
| WS1 | 09:00 | 0.295±0.025 bc | 123.931±10.442 c | 1.234±0.319 b | 8.941±1.207 d |
|  | 13:00 | 0.071±0.007 f | 53.754±5.518 e | 3.237±0.448 a | 67.210±9.669 b |
|  | 18:00 | 0.128±0.017 e | 53.770±7.139 e | 2.277±1.440 ab | 80.179±12.832 a |
| WS2 | 09:00 | 0.171±0.017 d | 71.863±7.110 de | 0.594±0.217 b | 27.989±3.199 c |
|  | 13:00 | 0.102±0.016 e | 76.843±12.422 d | 2.001±0.380 ab | 58.015±11.518 b |
|  | 18:00 | 0.122±0.018 e | 51.445±7.446 e | 1.513±0.696 ab | 71.769±4.939 a |
| Significance |  |  |  |  |  |
| Treat |  | <0.001 | <0.001 | 0.051 | <0.001 |
| Time |  | <0.001 | <0.001 | <0.001 | <0.001 |
| Treatment  * Time |  | <0.001 | <0.001 | 0.711 | <0.001 |

**Tab SM2.** Summary of the statistical analysis results for chlorophyll fluorescence parameters comparing well-watered plants (WW), water-stressed plants (WS1), and severely water-stressed plants (WS2) at four time points: 05:00 h, 09:00 h, 13:00 h, and 18:00 h (solar time). Tukey’s pairwise comparison was performed, and letters indicate significant differences among treatments (p < 0.05). Abbreviations are provided in the text.

| **Treat** | Time | **isoprene** |
| --- | --- | --- |
|  |  | **nmol m^-2^ s^-1^** |
| **WW** | 09:00 | 4.45 ± 1.9 d |
|  | 13:00 | 15.27 ± 2.61 c |
|  | 18:00 | 9.71 ± 2.40 cd |
| **WS1** | 09:00 | 2.63 ± 4.19 de |
|  | 13:00 | 30.36 ± 4.19 a |
|  | 18:00 | 22.25 ± 4.40 b |
| **WS2** | 09:00 | 1.83 ± 0.68 e |
|  | 13:00 | 12.28 ± 3.07 c |
|  | 18:00 | 9.82 ± 0.54 cd |
| **Significance** | | |
| Treatment |  | <0.001 |
| Time |  | <0.001 |
| Treatment*Time | | <0.001 |

**Tab SM3.** Summary of the statistical analysis results for isoprene emissions comparing well- watered plants (WW), water stress plants (WS1) and severe water stress plants (WS2) at three time points: 09:00 h, 13:00 h, and 18:00 h (solar time). Turkey pairwise comparison was performed, and letters indicate significant differences among treatments (*p* < 0.05). Abbreviations are shown in the text.

| **Treatment** | | | **Time** |  | **Treatment * Time** | |
| --- | --- | --- | --- | --- | --- | --- |
|  | F | p-value | F | p-value | F | p-value |
| Val | 87.897 | **<0.001** | 2.706 | 0.068 | 19.346 | **<0.001** |
| Thr | 47.071 | **<0.001** | 1.137 | 0.354 | 11.784 | **<0.001** |
| Gln | 44.671 | **<0.001** | 10.643 | **<0.001** | 9.074 | **<0.001** |
| Asn | 23.031 | **<0.001** | 11.641 | **<0.001** | 7.296 | **<0.001** |
| Chol | 15.085 | **<0.001** | 0.225 | 0.893 | 3.921 | **0.007** |
| Arg | 94.940 | **<0.001** | 3.285 | **0.038** | 8.201 | **<0.001** |
| Glu | 19.051 | **<0.001** | 12.310 | **<0.001** | 3.742 | **0.009** |
| Ala | 3.519 | **0.046** | 22.268 | **<0.001** | 11.608 | **<0.001** |
| MA | 17.419 | **<0.001** | 21.515 | **<0.001** | 9.973 | **<0.001** |
| AA | 5.027 | **0.015** | 0.178 | 0.910 | 1.346 | 0.276 |
| QA | 6.485 | **0.006** | 1.773 | 0.179 | 2.972 | **0.026** |
| SA | 11.150 | **<0.001** | 5.764 | **0.004** | 2.445 | 0.055 |
| CA | 17.912 | **<0.001** | 0.456 | 0.715 | 0.931 | 0.491 |
| Pyrocatechol | 0.512 | 0.606 | 1.117 | 0.362 | 1.379 | 0.263 |
| SalAlc | 78.204 | **<0.001** | 1.755 | 0.183 | 2.458 | 0.054 |
| Salicin | 1.671 | 0.209 | 1.139 | 0.353 | 2.532 | **0.048** |
| Salicortin | 1.263 | 0.301 | 6.226 | **0.003** | 1.063 | 0.411 |
| Rutin | 22.213 | **<0.001** | 4.357 | 0.014 | 9.183 | **<0.001** |
| GLC | 4.559 | **0.021** | 4.933 | **0.008** | 9.337 | **<0.001** |
| FRU | 2.271 | 0.125 | 3.289 | **0.038** | 1.006 | 0.444 |
| SUC | 344.576 | **<0.001** | 51.493 | **<0.001** | 32.617 | **<0.001** |
| MI | 16.728 | **<0.001** | 0.740 | 0.539 | 2.478 | 0.052 |

**Tab SM4.** Summary of the statistical analysis results for NMR-based leaf metabolomic data in well- watered plants (WW), water stress plants (WS1) and severe water stress plants (WS2) at four time points: 05:00 h, 09:00 h, 13:00 h, and 18:00 h (solar time). Tukey’s pairwise comparison was performed, and letters indicate significant differences among treatments (*p* < 0.05). Abbreviations: Valine (Val), Threonine (Thr), Glutamine (Gln), Asparagine (Asn), Choline (Chol), Arginine (Arg), Glutamic acid (Glu), Alanine (Ala), Malic acid (MA), Ascorbic acid (AA), Quinic Acid (QA), Succinic Acid (SA), Citric acid (CA), Pyrocatechol, Salicyl alcohol (SalAlc), Salicin, Salicortin, Rutin, Glucose (GLC), Fructose (FRU), Sucrose (SUC), Myo-inisitol (MI).

| **A)** |  |  |  |  |  |  |  |  |  |
| --- | --- | --- | --- | --- | --- | --- | --- | --- | --- |
|  |  | Val (µmol g^-1^  DW) | Thr (µmol g^-1^  DW) | Gln (µmol g^-1^  DW) | Asn (µmol g^-1^  DW) | Chol (µmol g^-1^  DW) | Arg (µmol g^-1^  DW) | Glu (µmol g^-1^  DW) | Ala (µmol g^-1^  DW) |
| WW |  | 0.53±0.26 b | 0.99±0.20 b | 5.56±1.87  b | 0.34±0.17  b | 1.24±0.33  b | 0.89±0.32  b | 13.88±1.56  b | 3.23±1.49  a |
| WS1 |  | 5.25±2.26 a | 3.92±1.95 a | 11.29±3.3  2 a | 3.50±3.00  a | 1.97±0.97  ab | 4.35±1.86  a | 22.98±6.21  a | 2.53±2.04  a |
| WS2 |  | 6.51±3.61 a | 5.30±2.68 a | 16.20±7.8  6 a | 7.65±7.51  a | 2.62±0.77  a | 6.74±2.17  a | 16.23±8.10  ab | 3.36±1.87  a |
| **B)** |  |  |  |  |  |  |  |  |  |
|  |  | Val  (µmol g^-1^ DW) | Thr  (µmol g^-1^ DW) | Gln  (µmol g^-1^ DW) | Asn  (µmol g^-1^ DW) | Chol  (µmol g^-1^ DW) | Arg  (µmol g^-1^ DW) | Glu  (µmol g^-1^ DW) | Ala  (µmol g^-1^ DW) |
| WW | 05:00 | 0.894  ±0.284 d | 0.978  ±0.127 c | 4.060  ±1.426 c | 0.321  ±0.210 c | 1.127  ±0.194 b | 1.117  ±0.209 d | 13.143  ±1.589 bc | 2.347  ±0.405 b |
|  | 09:00 | 0.383  ±0.079 d | 0.882  ±0.372 c | 7.582  ±1.31 bc | 0.499  ±0.086 c | 1.472  ±0.585 b | 0.920  ±0.267 d | 14.751  ±2.338 bc | 4.682  ±1.044 a |
|  | 13:00 | 0.409  ±0.167 d | 1.127  ±0.089 c | 6.241  ±0.837 c | 0.123  ±0.025 c | 1.251  ±0.279 b | 0.636  ±0.412 d | 14.281  ±0.130 b | 4.258  ±0.117 a |
|  | 18:00 | 0.447  ±0.038 d | 0.969  ±0.068 c | 4.369  ±1.544 c | 0.408  ±0.022 c | 1.122  ±0.079 b | 0.891  ±0.293 d | 13.332  ±1.637 c | 1.620  ±1.030 b |
| WS1 | 05:00 | 6.011  ±1.095 bc | 4.237  ±1.188 bc | 10.804  ±2.069 b | 6.092  ±3.543 b | 1.770  ±0.287 ab | 4.792  ±1.847 b | 19.362  ±2.176 b | 5.689  ±1.355 a |
|  | 09:00 | 2.394  ±0.695 d | 1.638  ±1.019 c | 10.715  ±2.333 b | 0.324  ±0.136 c | 0.856  ±0.301 b | 1.958  ±0.353 c | 15.305  ±1.465 b | 1.775  ±0.621 b |
|  | 13:00 | 7.625  ±0.695 b | 6.000  ±0.859 a | 13.462  ±4.242 b | 4.584  ±0.006 b | 2.569  ±0.228 a | 5.874  ±1.213 b | 28.594  ±0.254 a | 1.498  ±0.556 b |
|  | 18:00 | 4.952  ±1.96 c | 3.854  ±1.783 bc | 10.191  ±4.820 b | 3.003  ±2.945 bc | 2.668  ±1.329 a | 4.782  ±1.130 bc | 28.656  ±1.172 a | 1.174  ±0.406 b |
| WS2 | 05:00 | 7.055  ±1.912 b | 6.509  ±2.039 ab | 13.710  ±3.665 b | 18.306  ±7.507 a | 2.600  ±1.329 a | 8.192  ±2.417 a | 14.143  ±1.180 b | 6.134  ±0.299 a |
|  | 09:00 | 10.736  ±1.778 a | 7.941  ±1.929 a | 28.236  ±1.420 a | 4.700  ±1.287 bc | 3.353  ±0.854 a | 7.726  ±0.527 a | 7.656  ±3.846 c | 2.487  ±1.017 b |
|  | 13:00 | 1.691  ±1.313 cd | 1.796  ±0.576 c | 9.461  ±2.050 b | 1.198  ±0.280 c | 2.329  ±0.119 a | 3.661  ±0.128 c | 19.834  ±1.109 b | 2.909  ±1.725 ab |
|  | 18:00 | 6.542  ±1.415 cb | 4.936  ±0.489 b | 13.383  ±3.998 b | 6.393  ±1.983 b | 2.179  ±0.677 a | 7.375  ±0.487 a | 23.299  ±11.529 ab | 1.927  ±0.682 b |

**Tab SM5.** Quantification of amino acids identified by leaf NMR metabolomic profiling in well- watered plants (WW), water stress plants (WS1) and severe water stress plants (WS2) at four time points: 05:00 h, 09:00 h, 13:00 h, and 18:00 h (solar time). Tukey’s pairwise comparison was performed: A) letters indicate significant differences among treatments (*p* < 0.05), and B) letters indicate significant differences considering the interaction between treatment and sampling time (*p* < 0.05). Abbreviations: Valine (Val), Threonine (Thr), Glutamine (Gln), Asparagine (Asn), Choline (Chol), Arginine (Arg), Glutamic acid (Glu), Alanine (Ala).

| A) |  |  |  |  |  |  |
| --- | --- | --- | --- | --- | --- | --- |
|  |  | MA  (µmol g^-1^ DW) | AA  (µmol g^-1^ DW) | QA  (µmol g^-1^ DW) | SA  (µmol g^-1^ DW) | CA  (µmol g^-1^ DW) |
| WW |  | 15.50±4.56 ab | 20.49±3.29 ab | 66.86±9.09 a | 2.84±0.62 a | 11.84±2.26 a |
| WS1 |  | 13.57±3.27 b | 23.99±5.95 a | 57.06±16.96 ab | 2.12±0.80 b | 9.40±2.38 b |
| WS2 |  | 19.42±7.15 a | 18.53±2.79 b | 51.80±9.64 b | 1.83±0.68 b | 6.34±1.88 c |
| B) |  |  |  |  |  |  |
| Treat  ment | Time | MA  (µmol g^-1^ DW) | AA  (µmol g^-1^ DW) | QA  (µmol g^-1^ DW) | SA  (µmol g^-1^ DW) | CA  (µmol g^-1^ DW) |
| WW | 05:00 | 20.066±1.079 b | 22.869±2.334 ab | 68.243±12.563 ab | 3.228 ±0.690 a | 12.810±2.098 a |
|  | 09:00 | 16.760±0.151 c | 20.203±3.244 ab | 70.085±11.708 ab | 2.946±0.768 ab | 10.372±1.809 ab |
|  | 13:00 | 16.606±1.646 c | 20.875±3.801 ab | 68.291±8.432 ab | 2.850± 0.307 ab | 12.002±1.169 a |
|  | 18:00 | 8.582±0.367 d | 18.003±3.297 b | 60.829± 3.553 abc | 2.349±0.614 b | 12.164±3.773 a |
| WS1 | 05:00 | 17.357±0.759 c | 22.832±5.672 ab | 66.130±13.884 ab | 3.037±0.740 a | 10.043±2.622 ab |
|  | 09:00 | 13.287±1.881 d | 19.900±5.594 ab | 73.291±13.139 a | 1.470±0.532 c | 11.172±1.284 ab |
|  | 13:00 | 12.941±1.655 d | 23.328±4.660 ab | 47.497±8.629 bc | 1.965±0.684 bc | 7.909±1.448 ab |
|  | 18:00 | 10.704±1.373 d | 27.884±7.564 a | 41.335±10.790 c | 1.996±0.542 bc | 8.464±3.253 ab |
| WS2 | 05:00 | 16.816±2.180 c | 18.714±3.119 ab | 47.022± 5.827 c | 1.97±0.592 bc | 6.039±1.892 b |
|  | 09:00 | 30.258±4.871 a | 20.310±4.566 ab | 49.114±17.946 bc | 1.080±0.133 c | 7.333±1.762 b |
|  | 13:00 | 16.030±2.585 c | 17.789±2.717 ab | 50.981±0.236 bc | 2.707±0.139 ab | 6.532±2.532 b |
|  | 18:00 | 14.582±3.210 cd | 17.335±0.519 b | 60.08±2.578 bc | 1.561±0.135 bc | 5.445±2.586 b |

**Tab SM6.** Summary of the statistical analysis results of organic acids identified by leaf NMR metabolomic profiling in well-watered plants (WW), water stress plants (WS1) and severe water stress plants (WS2) at four time points: 5:00 h, 09:00 h, 13:00 h, and 18:00 h (solar time). Turkey pairwise comparison was performed: A) letters indicate significant differences among treatments (*p* < 0.05), and B) letters indicate significant differences considering the interaction between treatment and sampling time (*p* < 0.05). Abbreviations: Malic acid (MA), Ascorbic acid (AA), Quinic Acid (QA), Succinic Acid (SA), Citric acid (CA).

| **A)** |  |  |  |  |  |  |
| --- | --- | --- | --- | --- | --- | --- |
|  |  | **Pyrocatechol**  **(**µmol g^-1^ DW) | **SalAlc (**µmol  g^-1^ DW) | **Salicin**  **(**µmol g^-1^ DW) | **Salicort**  **(**µmol g^-1^ DW) | **Rutin**  **(**µmol g^-1^ DW) |
|  | WW | 23.11±6.20 a | 33.61±5.46 b | 62.04±10.66 a | 5.05±1.78 a | 11.75±1.91 b |
|  | WS1 | 21.23±4.01 a | 32.12±5.64 b | 69.24±16.95 a | 5.80±1.77 a | 12.89±1.32 b |
|  | WS2 | 22.79±4.68 a | 67.47±13.56 a | 59.61±17.24 a | 5.94±1.84 a | 14.64±1.38 a |
| **B)** |  |  |  |  |  |  |
| **Treatment** | **Time** | **Pyrocatechol**  **(**µmol g^-1^ DW) | **SalAlc (**µmol  g^-1^ DW) | **Salicin**  **(**µmol g^-1^ DW) | **Salicort**  **(**µmol g^-1^ DW) | **Rutin**  **(**µmol g^-1^ DW) |
| WW | 05:00 | 21.355 ± 6.399 a | 34.204 ±10.784 c | 60.550 ± 11.658 ab | 5.550 ± 1.473 ab | 11.031 ± 0.509 c |
|  | 09:00 | 25.730 ± 9.337 a | 31.850 ±1.463 c | 62.565 ± 6.400 ab | 3.732 ± 1.510 b | 14.569 ± 0.570 b |
|  | 13:00 | 25.560 ± 6.114 a | 30.918 ±1.994 c | 63.727 ± 16.924 ab | 5.000 ± 2.876 ab | 11.567 ± 0.834 c |
|  | 18:00 | 19.785 ± 2.336 a | 37.458 ±1.870 c | 61.312 ± 12.356 ab | 5.935 ± 0.677 ab | 9.822 ± 0.617 c |
| WS1 | 05:00 | 21.998 ±5.477 a | 32.427 ± 2.376 c | 72.639 ± 13.544 ab | 6.854 ± 0.789 ab | 12.267 ± 1.144 bc |
|  | 09:00 | 24.280 ±4.954 a | 30.444 ± 1.775 c | 83.506 ± 22.276 a | 5.294 ± 0.500 ab | 12.441 ±1.153 bc |
|  | 13:00 | 18.122 ±1.008 a | 35.078 ± 11.631 c | 61.993 ± 4.072 ab | 4.028 ± 0.765 ab | 14.352 ± 1.210 b |
|  | 18:00 | 20.526 ±1.732 a | 30.536 ± 3.090 c | 58.822 ± 17.877 ab | 7.016 ± 2.592 ab | 12.485 ± 1.065 bc |
| WS2 | 05:00 | 24.132 ±3.233 a | 79.477 ± 7.739 a | 69.042 ± 15.086 ab | 8.355 ± 0.406 a | 16.449 ± 0.747 a |
|  | 09:00 | 19.905 ±4.180 a | 70.276 ± 16.881 ab | 41.753 ± 7.555 b | 4.939 ± 1.293 b | 14.232 ± 1.41 b |
|  | 13:00 | 27.627 ± 3.366 a | 67.400 ± 14.305 ab | 75.913 ± 9.049 a | 4.364 ± 1.524 b | 13.595 ± 0.289 b |
|  | 18:00 | 19.485 ±4.501 a | 52.725 ± 3.049 bc | 51.720 ± 14.336 ab | 6.122 ± 1.289 ab | 14.208 ± 0.978 b |

**Tab SM7.** Summary of the statistical analysis results of phenolic compounds identified by leaf NMR metabolomic profiling in well-watered plants (WW), water stress plants (WS1) and severe water stress plants (WS2) at four time points: 5:00 h, 09:00 h, 13:00 h, and 18:00 h (solar time). Turkey pairwise comparison was performed: A) letters indicate significant differences among treatments (*p* < 0.05) and B) letters indicate significant differences considering the interaction between treatment and sampling time (*p* < 0.05). Abbreviations: Pyrocatechol, Salicyl alcohol (SalAlc), Salicin, Salicortin, Rutin.

| **A)** |  |  |  |  |  |
| --- | --- | --- | --- | --- | --- |
|  |  | **GLU**  (µmol g^-1^ DW) | **FRU**  (µmol g^-1^ DW) | **SUC**  **(**µmol g^-1^ DW) | **MI**  (µmol g^-1^ DW) |
|  | WW | 117.43±5.99 b | 44.31±12.19 a | 197.29±34.22 a | 47.71±3.97 b |
|  | WS1 | 121.93±18.96 ab | 36.26±12.69 a | 164.24±31.30 b | 54.21±6.83 a |
|  | WS2 | 128.99±19.36 a | 47.02±17.04 a | 94.61±33.23 c | 58.69±4.38 a |
| **B)** |  |  |  |  |  |
| **Treatment** | **Time** | **GLU**  (µmol g^-1^ DW) | **FRU**  (µmol g^-1^ DW) | **SUC**  **(**µmol g^-1^ DW) | **MI**  (µmol g^-1^ DW) |
| WW | 5:00 | 115.204 ± 10.961 c | 40.790 ± 9.721 ab | 146.083 ± 8.365 c | 49.455 ± 4.312 ab |
|  | 09:00 | 116.153 ±5.683 bc | 43.912 ± 11.830 ab | 211.945 ± 5.074 ab | 51.113 ± 2.307 b |
|  | 13:00 | 121.878 ±0.870 b | 51.280 ± 19.007 a | 228.721 ± 18.287 a | 46.608 ± 1.102 b |
|  | 18:00 | 116.488 ± 1.779 bc | 41.246 ± 10.760 ab | 202.396 ± 15.266 b | 43.678 ± 3.654 b |
| WS1 | 5:00 | 138.441 ± 10.354 b | 25.774 ± 11.811 b | 151.139 ± 11.479 c | 57.778 ± 5.603 ab |
|  | 09:00 | 134.771 ± 17.609 b | 39.373 ± 9.336 ab | 214.687 ± 4.481 a | 48.345 ± 8.844 b |
|  | 13:00 | 100.591 ± 5.597 c | 39.236 ± 11.977 ab | 142.848 ± 2.027 c | 51.389 ± 3.428 ab |
|  | 18:00 | 113.936 ± 9.612 cd | 40.656 ± 17.145 ab | 148.290 ± 9.498 c | 59.328 ± 3.633 a |
| WS2 | 5:00 | 133.951 ± 7.951 ab | 35.766 ±14.584 b | 56.012 ±3.537 d | 57.407 ±6.977 a |
|  | 09:00 | 120.488 ± 10.440 bc | 53.640 ±16.282 ab | 72.556 ±4.060 d | 61.297 ±5.317 a |
|  | 13:00 | 153.352 ± 18.685 a | 64.779 ±7.742 a | 131.039 ±6.681 c | 57.099 ±0.422 a |
|  | 18:00 | 108.157 ± 5.251 c | 33.881 ±10.080 b | 118.813 ±13.692 c | 58.954 ± 3.475 a |

**Tab SM8.** Summary of the statistical analysis results for sugars identified by leaf NMR metabolomic profiling in well-watered plants (WW), water stress plants (WS1) and severe water stress plants (WS2) at four time points: 5:00 h, 09:00 h, 13:00 h, and 18:00 h (solar time). Turkey pairwise comparison was performed: A) letters indicate significant differences among treatments (*p* < 0.05) and B) letters indicate significant differences considering the interaction between treatment and sampling time (*p* < 0.05). Abbreviations: Glucose (GLC), Fructose (FRU), Sucrose (SUC), Myo- inisitol (MI).

| Number of Components | Eigenvalue | Percent of Variance | Cumulative Percentage |
| --- | --- | --- | --- |
| 1 | 9.81599 | 42.678 | 42.678 |
| 2 | 4.18779 | 18.208 | **60.886** |
| 3 | 2.59259 | 11.272 | 72.158 |
| 4 | 2.18584 | 9.504 | 81.662 |
| 5 | 1.24602 | 5.417 | 87.079 |
| 6 | 0.940075 | 4.087 | 91.167 |
| 7 | 0.865534 | 3.763 | 94.930 |
| 8 | 0.516471 | 2.246 | 97.175 |
| 9 | 0.305357 | 1.328 | 98.503 |
| 10 | 0.252807 | 1.099 | 99.602 |
| 11 | 0.0915178 | 0.398 | 100.000 |
| 12 | 7.24418E-16 | 0.000 | 100.000 |
| 13 | 4.58391E-16 | 0.000 | 100.000 |
| 14 | 3.29245E-16 | 0.000 | 100.000 |
| 15 | 1.65761E-16 | 0.000 | 100.000 |
| 16 | 7.03065E-17 | 0.000 | 100.000 |
| 17 | 3.14259E-17 | 0.000 | 100.000 |
| 18 | 0.0 | 0.000 | 100.000 |
| 19 | 0.0 | 0.000 | 100.000 |
| 20 | 0.0 | 0.000 | 100.000 |
| 21 | 0.0 | 0.000 | 100.000 |
| 22 | 0.0 | 0.000 | 100.000 |
| 23 | 0.0 | 0.000 | 100.000 |

**Tab SM9.** Compositional Scree plot of the metabolites detected by NMR leaf metabolomic analysis. Each row reports the variance of the corresponding principal component.

|  | Component | Component 2 |
| --- | --- | --- |
| Val | 0.295783 | -0.0789839 |
| Thr | 0.304406 | -0.0556158 |
| Gln | 0.26878 | 0.0231411 |
| Asn | 0.233794 | 0.137447 |
| Chol | 0.294846 | -0.0681411 |
| Arg | 0.311773 | -0.0146526 |
| Glu | 0.0436672 | -0.335924 |
| Ala | 0.0247137 | 0.349864 |
| MA | 0.140441 | 0.206981 |
| AA | 0.00595376 | -0.324107 |
| Salicin | -0.144724 | 0.173097 |
| Salicortin | 0.109412 | 0.0694092 |
| SalAlc | 0.235458 | 0.253433 |
| Rutin | 0.234361 | 0.12052 |
| QA | -0.238491 | 0.154018 |
| SA | -0.203482 | 0.104854 |
| CA | -0.275337 | -0.0231537 |
| Pyrocatechol | -0.111932 | 0.355231 |
| GLC | -0.0128191 | 0.374848 |
| SUC | -0.290948 | -0.0821753 |
| FRU | -0.0147371 | 0.109535 |
| MI | 0.267485 | 0.0436709 |
| ISO | 0.00702935 | -0.383541 |

**Tab SM10.** Specific loadings of the metabolites detected by NMR leaf metabolomic analysis.
